# The impact of regional STEMI systems on protocol use and quality improvement initiatives in community hospitals without cardiac catheterization laboratories

**DOI:** 10.1016/j.ahjo.2021.100077

**Published:** 2021-12-09

**Authors:** Chauncy B. Handran, Miranda Kunz, David M. Larson, Ross F. Garberich, Kelsey Baran, Jason T. Henry, Scott W. Sharkey, Timothy D. Henry

**Affiliations:** a Prisma Health, Greenville, SC, United States of America; b Minneapolis Heart Institute Foundation at Abbott Northwestern Hospital, Minneapolis, MN, United States of America; c Ridgeview Medical Center, Waconia, MN, United States of America; d Berkshire Medical Center, Pittsfield, MA, United States of America; e Sarah Cannon Research Institute at HealthONE, Denver, CO, United States of America; f The Carl and Edyth Lindner Center for Research and Education, The Christ Hospital, Cincinnati, OH, United States of America

**Keywords:** STEMI, Regional Systems of Care, ACS, PCI

## Abstract

**Study objective:**

Since the 1990s, national guidelines have recommended hospitals develop STEMI treatment protocols and monitor quality. A 2003 survey of Minnesota hospitals without cardiac catheterization laboratories (CCL) found <2/3 had STEMI protocols, <50% had a quality assessment (QA) process, and protocols in existence were incomplete. We evaluated temporal changes in STEMI processes in relationship to changes in mortality.

**Design, setting, and participants:**

Follow-up surveys were mailed to emergency departments at 108 Minnesota hospitals without CCL.

**Results:**

Among 87% of responding hospitals, 89% had formal protocols or guidelines for STEMI management compared to 63% in 2003 (*p* < 0.001). In 2010, 67% of hospitals had triage/transfer criteria and 15% of hospitals used protocols for transfer decisions, compared to only 8% (p < 0.001) and 1% (*p* = 0.098), respectively, in 2003. The percentage of hospitals transferring patients with STEMI from the emergency department increased from 23% in 2003 to 56% in 2010 (*p* < 0.001). During this time, age-adjusted acute MI mortality rate in Minnesota decreased 33% and was more pronounced in areas with regional STEMI systems.

**Conclusions:**

Since 2003, utilization of STEMI guidelines, protocols, and standing orders in Minnesota hospitals without CCL has markedly improved with <10% of hospitals lacking specific STEMI management protocols. The majority of hospitals routinely transfer patients with STEMI for primary PCI and have comprehensive QA processes. This improvement was stimulated by regional STEMI systems, further supporting the current class I recommendation for STEMI systems of care in current guidelines. The decline in Minnesota STEMI mortality paralleled the growth of regional STEMI systems.

## Graphical abstract

**Figure:**
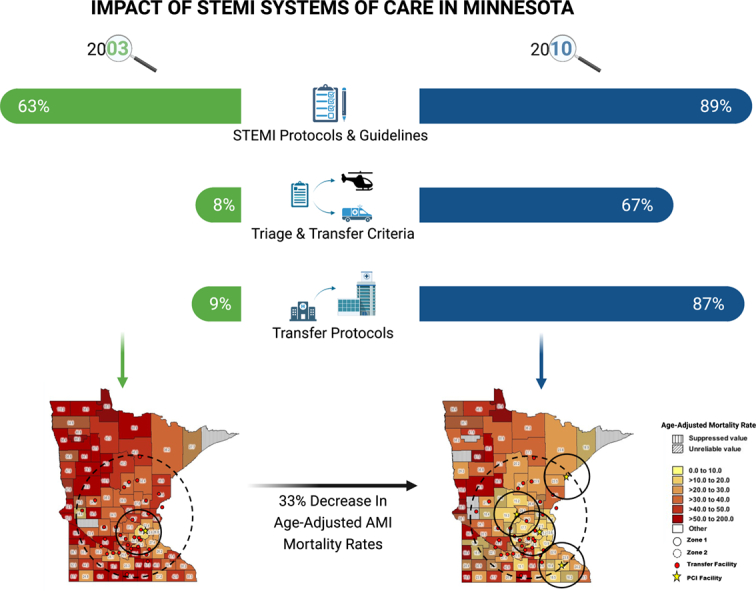

## 1 Introduction

Although there have been significant advances in the diagnosis and management of ST-segment myocardial infarction (STEMI), implementation of those into clinical practice is at times inconsistent [1].Primary percutaneous coronary intervention (PCI) is well established as the preferred reperfusion strategy for STEMI [1], [2], [3], [4]. Nonetheless, disparities remain in the United States (US) as many patients do not have timely access to PCI, particularly in rural areas [5], [6]. Nearly thirty years ago, the National Heart Attack Alert Program recommended all emergency departments develop STEMI protocols and monitor quality measures such as time to reperfusion [7]. However, a 2003 Minnesota survey study found 33% of non-PCI capable hospitals did not have any guidelines, protocols, or standing orders for STEMI treatment, and others had incomplete or inadequate STEMI specific guidelines [8]. Furthermore, only 50% of non-PCI facilities utilized a formal quality assessment process and outcomes were inconsistently evaluated.

In response to these findings, the authors developed an integrated STEMI system of care that included the Minneapolis Heart Institute (MHI) (PCI center) and 31 non-PCI referring hospitals [9], [10], [11], [12]. Key components of this system included development and implementation of treatment and transfer protocols and assisting the referring hospitals with quality improvement measures. Other Minnesota PCI centers quickly followed this lead and implemented their own STEMI referral systems [13], [14], [15].

Subsequently, the American Heart Association (AHA) has also promoted the development of STEMI systems of care through its Mission Lifeline initiative in order improve quality of care and timely access to PCI [16], [17], [18], [19], [20]. The Updated 2009 AHA and American College of Cardiology (ACC) STEMI Guidelines recommended STEMI systems of care as a class I indication [3].

The objective of this study was to assess the impact of the development of regional STEMI systems on the use of protocols, guidelines, quality assessment methods, and decision-making regarding treatment and transfer criteria for patients with STEMI in Minnesota non-PCI hospitals.

## 2 Materials and methods

### 2.1 Study design

This was a serial cross-sectional survey study to determine the evolution of hospital-specific protocols and guidelines for treatment of patients with STEMI. These outcomes were obtained and assessed by comparing the results of two surveys conducted in 2003 and 2010 [8]. Institutional Review Board approval was obtained for data collection, follow-up, and data analysis.

### 2.2 Survey content and administration

All Minnesota community hospitals without primary PCI capability had surveys sent to emergency department (ED) medical directors and nurse managers in 2003 (111 hospitals) and 2010 (108 hospitals). Surveys included questions regarding protocols, standing orders, decision-making, quality assurance, hospital size, distance to nearest PCI facility, and indications for Patients with STEMI transfer (Appendix A). All 2003 survey content was included in the 2010 survey with the addition of indications for transfer for primary PCI and decision-making for PCI activation and transfer initiation. Hospitals not initially responding to the survey were sent a second letter, followed by a phone call. All 108 hospitals were included in the original survey which also included 3 hospitals that subsequently closed.

### 2.3 Statewide acute myocardial infarction (AMI) mortality data

For Minnesota, statewide and county-level age-adjusted AMI death rates and maps were obtained from the Center for Disease Control Wide-ranging ONline Data for Epidemiologic Research (CDC WONDER) database [21]. Maps were superimposed with the locations of PCI-capable facilities, transfer facilities, and transfer zones.

### 2.4 Statistical analysis

Continuous variables were expressed as the mean ± SD (range), and discrete variables and categorical data were expressed as frequencies and percentages. For comparison of the 2010 and 2003 surveys, analyses were conducted using Pearson's Chi-Squared or Fisher's Exact Tests. Two-tailed *p*-values were reported in analyses and *p* < 0.05 was used to assess statistical significance. All analyses were performed with Stata Version 16.2 (StataCorp, College Station, TX).

## 3 Results

Among Minnesota non-PCI hospitals, the survey response was similar with 94/108 (87%) in 2010 and 104/111 (94%) in 2003. The surveys were completed by ED medical directors at 31 (33%) hospitals, by ED nurse managers at 44 (47%) hospitals, by both at 17 (8%) hospitals, and by unspecified personnel at 2 (2%) hospitals.

### 3.1 STEMI protocols and guidelines

**Fig. 1:**
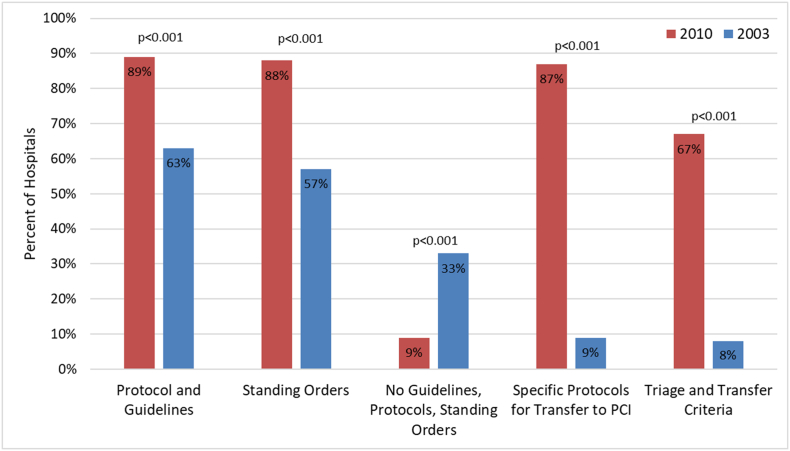
Guidelines, protocols, standing orders comparison from 2003 to 2010.

Protocols and guidelines from the 2010 and 2003 surveys are compared in Fig. 1 and Table 1. In 2010, 89% of hospitals had protocols or guidelines and 88% had standing orders, compared to 63% and 57%, respectively, in 2003 (*p* < 0.001). In 2010, only 9% of hospitals had no protocols, guidelines, or standing orders, compared to 33% in 2003 (*p* < 0.001). In 2010, 87% of hospitals with guidelines, protocols, and standing orders addressed criteria to transfer to a PCI facility, whereas only 9% did so in 2003 (p < 0.001). Current protocols are more detailed and specific regarding indications for adjunctive medications. For example, in 2010 there was a significant increase in protocols defining indications for aspirin use (99% vs 91%, *p* = 0.027), beta-blockers (93% vs 71%, *p* < 0.001), intravenous nitroglycerin (85% vs 59%, p < 0.001) and unfractionated heparin (94% vs 80%, *p* = 0.008) compared to the 2003 survey.

**Table 1:** Protocols, guidelines, standing orders comparison between 2010 and 2003.

	2010 Responses	2010 n value	2003 Responses	2003 n value	*P* value
Written protocol and guideline, (%)	83 (89)	93	65 (63)	104	<0.001
Standing orders, (%)	83 (88)	94	59 (57)	104	<0.001
Without guideline, protocol, and standing orders^a^, (%)	8 (9)	94	34 (33)	104	<0.001
Protocol specific indications for transfer for primary PCI^a^, ^b^, (%)	72 (87)	83	6 (9)	70	<0.001
Thrombolytics, (%)	72 (89)	81	60 (86)	70	0.558
Aspirin, (%)	84 (99)	85	64 (91)	70	0.027
Beta-blockers, (%)	79 (93)	85	50 (71)	70	<0.001
Intravenous nitroglycerin, (%)	70 (85)	82	41 (59)	70	<0.001
IV^c^ unfractionated heparin	79 (94)	84	56 (80)	70	0.008
Triage and transfer criteria	56 (67)	84	6 (8)	70	<0.001
Formal quality assessment process, (%)	66 (71)	93	52 (50)	104	0.003

a Tests done using Fischer's exact test.

b PCI = percutaneous coronary intervention.

c IV = intravenous.

### 3.2 Decision-making and transfer issues

**Fig. 2:**
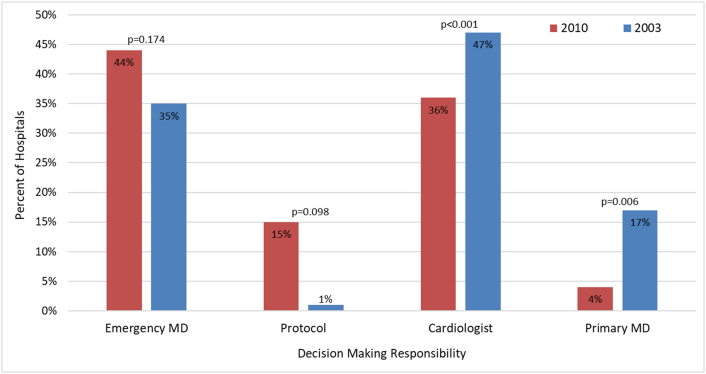
Decision making responsibility from 2003 to 2010.

The survey queried hospitals regarding decision-making processes involving transfer and reperfusion therapy. Fig. 2 represents the comparison of transfer and decision-making between 2010 and 2003. In 2010, before transferring patients directly from the ED to a tertiary hospital only 4% (4/93) of emergency physicians required discussion with the admitting (attending) physician compared to 17% (18/104) in 2003 (*p* = 0.006). In 2010, the transfer decision was made exclusively by written protocol in 14/93 (15%) hospitals vs 1/104 (1%) in 2003 (*p* = 0.098). In 2010, an estimated 68% of patients with STEMI were transferred compared to 32% in 2003 (*p* < 0.001). Additionally, direct transfer of patients with STEMI from the ED occurred in 56% of responding hospitals in 2010 vs. 23% in 2003 (*p* < 0.001).

### 3.3 Quality assurance

**Fig. 3:**
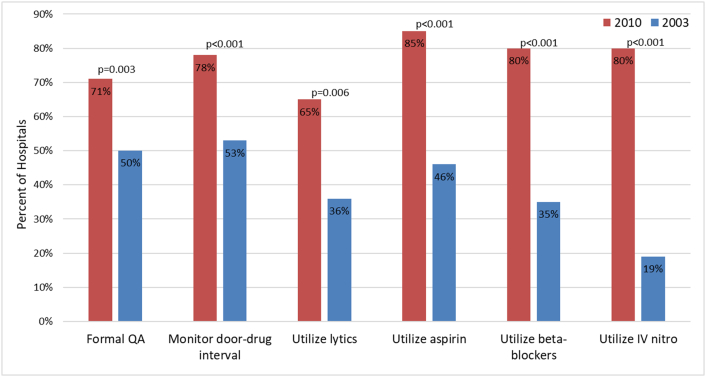
Quality assurance, monitor door-drug intervals comparison from 2003 to 2010.

Differences in quality assessment processes from 2010 to 2003 are shown in Fig. 3. In 2010, 66/93 (71%) surveyed hospitals reported formal quality assessment processes vs. 52/104 (50%) (*p* = 0.003) in 2003. Quality assessment measurement parameters significantly improved in the 2010 survey: 78% documented door-to-drug intervals and drug administration including thrombolytic therapy (65%), aspirin (85%), beta-blockers (80%) and intravenous nitroglycerin (80%). These results are significantly improved in each category compared to the 2003 survey (Fig. 3).

### 3.4 AMI mortality

**Fig. 4:**
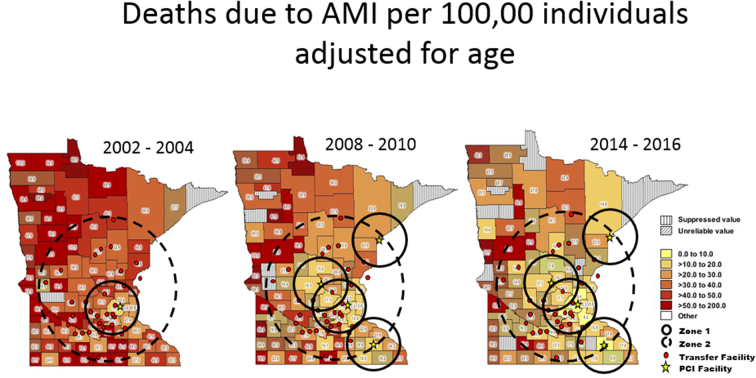
Deaths due to AMI per 100,000 individuals, adjusted for age 2002–2004 and 2008–2010.

Between 2003 and 2010, the Minnesota age-adjusted AMI mortality decreased by 33%, decreases which was most notable in those counties within transfer distance of the MHI STEMI system (Fig. 4). Counties within the MHI transfer zone 2 experienced an average decrease in age-adjusted mortality rate of 36% between 2003 and 2010. In comparison, counties outside zone 2 experienced only an average 25% decrease in mortality rate (*p* = 0.029). Over the 12 years between 2004 and 2016, statewide age-adjusted acute myocardial infarction mortality decreased by 46%. This decrease was more pronounced for patients presenting <60 miles from a PCI-capable facility than for those presenting >60 miles from a PCI-capable facility (58% vs. 38%, p < 0.001). The growth of other organized STEMI systems in the state likely account for some of the decreases in morality as well [13], [14], [15].

## 4 Discussion

Since implementation of a regional STEMI system throughout Minnesota in 2003, the use of guidelines and specific protocols to make treatment and transfer decisions has improved significantly. More hospitals now have guidelines, protocols, and standing orders for the treatment of patients with STEMI compared to 2003 and transfer decisions are now being made more often by emergency physicians than by cardiologists or primary physicians as recommended by current guidelines [3], [4].

Timely treatment and transfer decisions are vital as there is unequivocal evidence that primary PCI is superior to fibrinolytic therapy alone in reducing mortality and major adverse cardiac events in patients with STEMI [1], [2], [3], [4]. However, among 4050 US acute-care centers, only ~1500 are PCI-capable, making it challenging to provide timely primary PCI for STEMI treatment [5], [6]. In fact, >40 million US adults live further than 60 min from a PCI-capable facility, and 25% of the population requires >30 min to reach a primary PCI facility. Consequently, the 2009 focused update of the ACC/AHA guidelines for STEMI management recommended implementation of hospital-specific guidelines, protocols, and standing orders to reduce the variability in care and reduce the time to perfusion of the infarct related artery [3]. Barriers to efficient transfer of patients with STEMI from non-PCI capable hospitals include diagnostic uncertainty, non-uniform health systems integration, lack of inter-facility transfer protocols (non-PCI center to PCI center), 24-hours-a-day/7-days-a-week PCI center staffing coverage (interventional cardiologists and technical staff), and reimbursement policies [1].

To address these obstacles, MHI established the Level 1 Program, a regional STEMI system of care that has evolved into a 32-center standardized Minnesota system with the goal of quality care improvement throughout the region. Between January 2003–August 2018, the formation of a rapid response 24/7 on-call catheterization laboratory team at MHI allowed rapid PCI treatment for 5858 patients, with 4088 (70%) of these patients transferred from the 31 Level 1 trained non-PCI capable facilities. The Level 1 STEMI program provides training tools for community hospitals and transport personnel through a series of educational/training programs for ED nurses and personnel, ED physicians, and primary care physicians along with educational digital content provided for each site [9], [10], [11], [12]. Cardiologists, emergency physicians, nurses, paramedics, transport personnel, and clinical researchers at MHI collaborate to develop appropriate changes in protocols and guidelines to address problems encountered during the transfer process for specific hospitals [9], [10], [11], [12]. To revise and instruct new protocols, the Level 1 program employs a cardiovascular emergency coordinator who travels to 31 community hospitals to verify appropriate use of guidelines, protocols, and standing orders and to provide training as needed. MHI also utilizes a Level 1 database coordinator to populate reports with time to treatment, clinical characteristics, and 5-year outcomes. This data collection allows for rapid-cycle quality assessment, which is then reported back to referring hospitals, allowing for continuous improvement in inter-facility transfers and most importantly the integration of a standardized system of care [9], [10], [11], [12]. Of note, the majority of the non-PCI centers do not have on-site cardiologists but MHI (as well as the other large regional STEMI systems) have robust cardiology outreach programs [8], [9], [10], [11], [12], [13], [14], [15]. Therefore, the majority of the subsequent follow up care is provided in the local community.

Level 1 affiliated hospital personnel have biannual meetings with physician and nurse representatives from each community hospital to discuss problems and outcomes. Door-to-balloon times and CCL to balloon times for each individual interventional cardiologist are reviewed on a regular basis [9], [10], [11], [12]. In addition, MHI developed a mobile application which includes details of the standardized protocols.

A comparison of 2010 and 2003 surveys verify that Minnesota non-PCI centers improved their respective STEMI procedures through the utilization of an organized system of care provided by the MHI and other regional STEMI systems [13], [14], [15]. Although only 31 hospitals participate in the Level 1 program statewide, the majority of the other 77 non-PCI hospitals are part of other organized regional systems to transfer patients with STEMIs efficiently [13], [14], [15]. Reducing the variability of care and implementing evidence-based clinical protocols with a well-coordinated STEMI system of care is associated with significant improvements in morbidity and mortality, as reflected in the significant improvement in mortality noted in Minnesota which closely tracks the growth of regional STEMI systems [9], [10], [11], [12], [13], [14], [15].

There is general consensus that each US hospital should have a detailed plan regarding STEMI care which includes individual outcome information [1], [9]. Our survey indicates the significant progress made by Minnesota regional STEMI systems was associated with an important reduction in mortality.

### 4.1 Limitations

These results reflect self-reported surveys; therefore, we are unable to verify the decision process for patient transfer at each site. Individual factors involving patient presentation or transport, which existing guidelines do not address, may have influenced the treatment and transfer decision-making. The survey data provides an estimate regarding community hospital guideline implementation and quality of performance consequent to participation in an organized system.

With the development and growth of regional STEMI systems, more than 90% of community hospitals in Minnesota implemented guidelines and standardized protocols for STEMI care. These regional STEMI systems played a key role in the significant reduction in cardiovascular mortality in the state of Minnesota.

## Declaration of competing interest

The authors declare that they have no known competing financial interests or personal relationships that could have appeared to influence the work reported in this paper.

## Appendix A Supplementary data

Appendix A: The 2010 survey sent to 108 non-PCI community hospitals throughout the state of Minnesota. Image 1
