# Supplementary material for: The impact of regional STEMI systems on protocol use and quality improvement initiatives in community hospitals without cardiac catheterization laboratories
Source: Am Heart J Plus. 2021 Dec 9;13:100077. doi: 10.1016/j.ahjo.2021.100077 (PMC10978212; doi:10.1016/j.ahjo.2021.100077)
Supplement: Supplementary file 1 — Appendix A: The 2010 survey sent to 108 non-PCI community hospitals throughout the state of Minnesota. [file mmc1.docx]

Appendix A: The 2010 survey sent to 108 non-PCI community hospitals throughout the state of Minnesota.

**Survey of Community Hospitals regarding the treatment of Acute ST Elevation Myocardial Infarctions**

- ***Acute ST Elevation Myocardial Infarctions only***
- ***This does not apply to patients with unstable angina or patients with chest pain without diagnostic ST Elevation***
- ***This information may be anonymous and will be kept confidential.***

1. Do you have a written protocol or guideline **in the Emergency department** (written specifically for your hospital) regarding the management of Acute ST Elevation Myocardial Infarction? *Yes No*
2. Do you have standing orders for Acute STEMI that are used in the Emergency Department? *Yes No*
3. If no to 1 ***and*** 2, go to 6.
4. Do these protocols, guidelines or standing orders address the following:
   1. Indications for transfer for primary PCI? *Yes No*
   2. Indications for fibrinolytic therapy? *Yes No, NA*
   3. Which fibrinolytic to use and dose? *Yes No, NA*
   4. Indications and dose for Beta blockers? *Yes No*
   5. Indications and dose for Aspirin?  *Yes No*
   6. Indications and dose of anti-platelet therapy (e.g. Clopidogrel or GP IIb/IIIa inhibitor)? *Yes No*
   7. Indications and dose of IV unfractionated heparin? *Yes No*
   8. Indications and dose of intravenous nitroglycerin? *Yes No*
5. Does your protocol define the disposition of Acute MI patients. (e.g. which patients are admitted vs transferred to a tertiary facility from the Emergency department)? *Yes No*
6. The decision to activate the catheterization lab at the PCI hospital and initiate the transfer protocol is **most often** made by: (circle one)
   1. Emergency department physician (the first physician seeing the patient in the ED) independently
   2. The Emergency department physician by following a written protocol in the ED
   3. The Emergency department physician only after telephone consultation with the admitting (attending) physician
   4. The Emergency department physician only after telephone consultation with a cardiologist.
7. The decision to administer a thrombolytic (fibrinolytic) in your ED is ***most often*** made by: *(circle one)*
   1. Emergency department physician (the first physician seeing the patient in the ED) independently
   2. The Emergency department physician by following a written protocol in the ED
   3. The Emergency department physician only after telephone consultation with the admitting (attending ) physician
   4. The Emergency department physician only after telephone consultation with a cardiologist.
8. The decision to transfer the patient directly from the Emergency Department to a tertiary facility is ***most often*** made by: *(circle one)*
   1. Emergency department physician (the first physician seeing the patient in the ED) independently
   2. The Emergency department physician by following a written protocol in the ED
   3. The Emergency department physician only after telephone consultation with the admitting (attending ) physician
   4. The Emergency department physician only after telephone consultation with a cardiologist
9. When transferring a patient with an Acute STEMI from your ED, what is your preferred mode of transfer (assuming good weather conditions)? *(circle one)*
   1. Helicopter
   2. Ground Critical care (Critical Care RN and paramedic)
   3. Ground ALS (paramedics)
   4. Ground BLS (EMTs) with RN from your hospital
10. Does your hospital have a formal Quality Assessment process that looks at all acute ST elevation Myocardial infarctions? *Yes No*
11. Does this QA process report data on the following:
    1. In door-out door time in transferred patients? *Yes No*
    2. Door to drug intervals (when giving fibrinolytics)? *Yes No*
    3. Percent of patients getting fibrinolytics in the ED? *Yes No*
    4. Percent of patients given Aspirin in the ED? *Yes No*
    5. Percent of patients given beta blockers in ED? *Yes No*
    6. Percent of patients given IV Nitro in the ED? *Yes No*
12. Is there a physician that oversees the QA process? *Yes No*
13. If yes, what physician oversees the QA process? *(circle one)*
    1. Emergency department Medical Director
    2. Chair of Internal Medicine or Family Practice
    3. Chief of Staff
    4. Hospital Medical Director
    5. Other _______________________
14. How many Acute ST Elevation Myocardial Infarctions were seen in your Emergency Department in the last 12 months? ____________
15. In patients with Acute ST elevation MI under the age of 75, what percent were transferred to a tertiary hospital for specialized cardiovascular care? (circle one)
    1. 100%
    2. 90-100%
    3. 75-90%
    4. 50-75%
    5. less than 50%
16. Of the patients transferred ( # 14 ), what percent were transferred directly from the ED? *(circle one)*
    1. 100%
    2. 90-100%
    3. 75-90%
    4. 50-75%
    5. less than 50%
17. What is the approximate distance (miles) to the tertiary hospital that you most often refer Acute MI patients to? ______________
18. How many licensed beds does your hospital have? ___________
19. How many ED visits per year? _____________
20. Would you be interested in participating in a multicenter Community hospital research project looking at ways to improve the management of Acute Myocardial Infarctions? *Yes No*
21. If yes,
    1. Physician contact: _________________________
    2. Nurse contact: ____________________________

Name of your hospital _________________________ (optional)

Your name _______________________________(optional)
